# Probable Cerebral Amyloid Angiopathy-Related Inflammation Presenting as an Incidental MRI Finding in an Elderly Patient: A Case Report

**DOI:** 10.7759/cureus.84282

**Published:** 2025-05-17

**Authors:** Tatsuya Tanaka, Takashi Muto, Taku Goto, Shiki Nakayama, Akira Matsuno

**Affiliations:** 1 Department of Neurosurgery, International University of Health and Welfare Narita Hospital, Narita, JPN; 2 Department of Nephrology, Kouhoukai Takagi Hospital, Okawa, JPN; 3 Department of Emergency Medicine, Kouhoukai Takagi Hospital, Okawa, JPN

**Keywords:** asymptomatic, cerebral amyloid angiopathy, cerebral amyloid angiopathy-related inflammation (caa-ri), cognitive decline, conservative management, head trauma, microbleeds

## Abstract

Cerebral amyloid angiopathy-related inflammation (CAA-ri) is a rare but treatable cause of subacute cognitive decline and neurological dysfunction, particularly in the elderly.

We report the case of an 88-year-old woman with mild dementia who was independent in her activities of daily living. She presented after minor head trauma, and brain MRI revealed multiple cerebral microbleeds in the bilateral cortices, cerebellum, and thalamus, along with diffuse fluid-attenuated inversion recovery (FLAIR) hyperintensities, suggestive of probable CAA-ri. Given the absence of symptoms, a strategy of close observation was initially adopted. However, two months later, she developed decreased appetite and gait instability, followed by a transient loss of consciousness at three months, necessitating hospitalization. During admission, she subsequently developed disuse syndrome, resulting in discharge to a nursing facility. Follow-up MRI at six months showed resolution of the prior FLAIR hyperintensities but revealed prominent medial temporal lobe atrophy, supporting the diagnosis of probable CAA-ri.

This case underscores that even in asymptomatic patients with significant MRI findings consistent with CAA-ri, there is a considerable risk of neurological deterioration and functional decline. While some mild cases may experience spontaneous resolution, the presence of widespread edema and multiple microbleeds may necessitate early immunosuppressive intervention, even if the patient is asymptomatic. The clinical silence of asymptomatic CAA-ri should not lead to an underestimation of its potential for progression. A timely assessment of risk and intervention may be crucial to prevent irreversible neurological and functional impairment.

## Introduction

Cerebral amyloid angiopathy (CAA) is a major cause of cortical and microbleeds in the elderly and is also associated with Alzheimer's disease, making it a significant neurovascular disorder [1]. One subtype of CAA, known as cerebral amyloid angiopathy-related inflammation (CAA-ri), is characterized by inflammatory changes around blood vessels, which are a result of an immune response to amyloid-β deposition [2,3].

CAA-ri can present with a variety of clinical manifestations, including acute or subacute headache, cognitive decline, focal neurological deficits, and seizures. However, with the recent advancements in magnetic resonance imaging (MRI) technology, asymptomatic cases of CAA-ri have been incidentally detected [3-5]. The natural history of asymptomatic CAA-ri is still not well understood. Some cases may resolve spontaneously, while others may progress to symptomatic disease and irreversible brain dysfunction if left untreated. Therefore, clear management guidelines for these cases remain uncertain [4,6,7].

This report describes a case of probable asymptomatic CAA-ri, which was incidentally discovered after head trauma. Although brain biopsy was not performed, the diagnosis of probable CAA-ri was made based on the Modified Boston Criteria, which incorporates characteristic MRI findings, notably fluid-attenuated inversion recovery (FLAIR) hyperintensities and multiple cerebral microbleeds, along with clinical context. [3]. Initially, the patient was managed with observation, but later developed decreased appetite, falls, and fractures, leading to a decline in functional status. Through this case, we discuss the characteristics of the natural course of asymptomatic CAA-ri and emphasize the importance of early risk assessment and management.

## Case presentation

An 88-year-old woman with mild dementia, but independent in her activities of daily living, presented to our hospital after a fall at home, which resulted in a head trauma. Upon arrival, her vital signs were as follows: temperature 37.4 °C, blood pressure 187/95 mmHg, pulse rate 74 beats per minute, and SpO₂ 97% (room air). Her Glasgow Coma Scale (GCS) score was 14 (E4V4M6). Bruising and swelling were noted around her right eye, and abrasions and contusions were observed on her limbs; however, there were no signs of limb paralysis or paresthesia. Eye movements, facial movements, and tongue movements were all normal.

**Figure 1:**
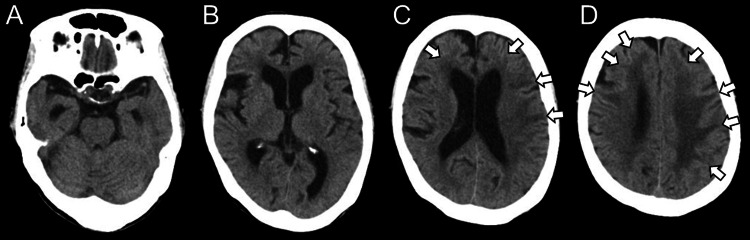
Initial head CT Axial views at the level of the brainstem (A), basal ganglia (B), parietal lobes (C), and frontal lobes (D). Head CT revealed no traumatic intracranial hemorrhage or skull fractures. Multiple hypodense areas were observed in both cerebral hemispheres (arrows).

Head computed tomography did not reveal any intracranial hemorrhage or fractures related to the trauma, but multiple areas of hypodensity were observed in both cerebral hemispheres (Figure 1).

**Figure 2:**
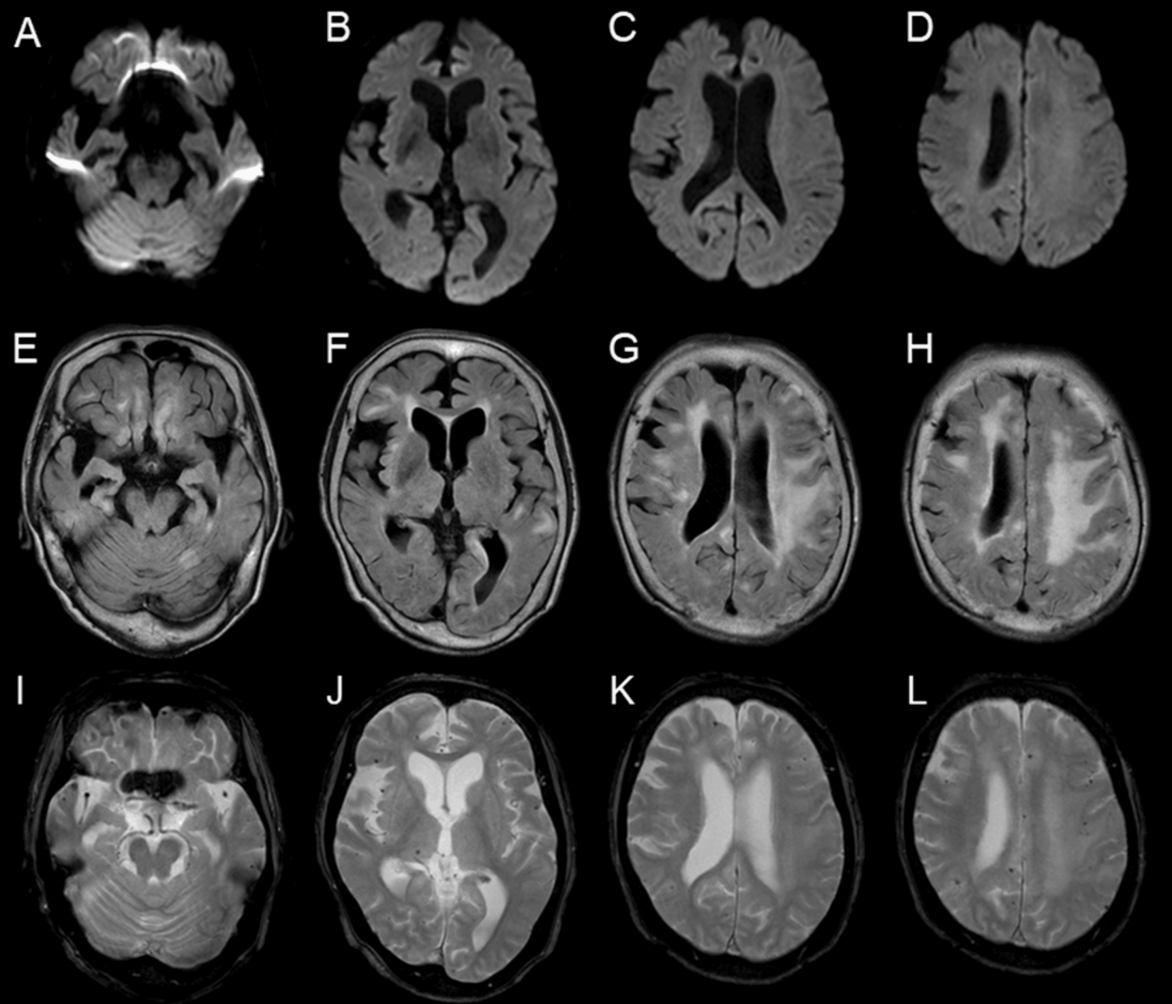
Initial head MRI (A–D) Diffusion-weighted imaging (DWI); (E–H) Fluid-attenuated inversion recovery (FLAIR); (I–L) T2*-weighted imaging DWI showed no abnormalities. FLAIR images demonstrated multiple hyperintense lesions in the cerebellum and bilateral cortical regions. T2*-weighted images revealed multiple cerebral microbleeds in the right thalamus and bilateral cortical and subcortical areas.

Head MRI demonstrated multiple microbleeds in the cerebellum, right thalamus, bilateral cortical, and subcortical areas. FLAIR imaging showed high-signal areas consistent with edema in the bilateral cerebral cortex, subcortical areas, and cerebellum, with extensive involvement across multiple brain regions (Figure 2).

The differential diagnosis included various forms of encephalitis and encephalopathy, including CAA-ri. MR angiography revealed a 2-mm unruptured cerebral aneurysm in the left middle cerebral artery.

Initially, due to the absence of symptoms, the decision was made to perform further investigations if symptoms developed, with a follow-up examination scheduled in six months. However, two months later, the patient showed decreased appetite and an increased tendency to fall. Three months later, she experienced a transient loss of consciousness and presented to the emergency department, where she was diagnosed with hypokalemia and subsequently admitted. During hospitalization, she developed a COVID-19 infection and presented with disuse syndrome, leading to her discharge to a nursing facility. Five months later, she sustained a fracture of the right femoral neck due to another fall and underwent orthopedic surgery for fracture fixation.

**Figure 3:**
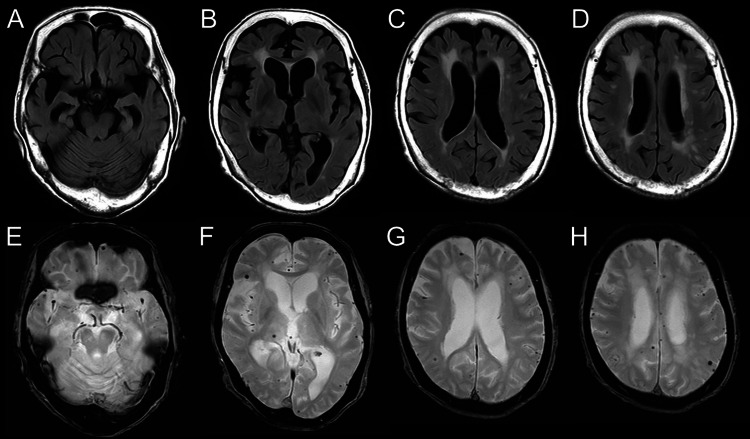
Follow-up head MRI at 6 months (A–D) Fluid-attenuated inversion recovery (FLAIR); (E–H) T2*-weighted imaging FLAIR hyperintense lesions previously observed in the cerebellum and bilateral cerebral white matter had resolved. However, multiple cerebral microbleeds persisted in the right thalamus and bilateral cortical and subcortical regions.

Six months later, follow-up MRI of the head showed resolution of the previously observed FLAIR high-signal areas. However, significant brain atrophy was noted in the bilateral medial temporal lobes (Figure 3).

Based on these findings and according to established diagnostic criteria [3], the patient was diagnosed with probable CAA-ri.

## Discussion

This case report presents a rare instance of asymptomatic CAA-ri incidentally identified following head trauma, which gradually progressed to symptomatic disease and functional decline. It highlights the challenges of managing probable CAA-ri that initially lacks overt symptoms but exhibits extensive MRI abnormalities, including widespread white matter edema and cortical microbleeds. While some asymptomatic cases may resolve spontaneously, others, as in this case, can experience delayed neurological deterioration, underscoring the importance of early risk stratification and timely consideration of immunosuppressive therapy.

Some asymptomatic cases of CAA-ri have been reported to resolve spontaneously. Auriel et al. observed that in asymptomatic CAA-ri patients, careful observation without immunotherapy led to the spontaneous resolution of MRI abnormalities [3]. Additionally, spontaneous improvement has been noted in cases of amyloid-related imaging abnormalities (ARIA)-like events without treatment [4]. On the other hand, the risk of symptom development remains in asymptomatic CAA-ri, even when untreated. Particularly in cases with imaging findings, such as widespread white matter edema, multiple cortical microbleeds (CMBs), and cortical superficial siderosis (cSS), the risk of progression to neurological symptoms, cognitive decline, and even fatal brain hemorrhage is considered high [7].

In the present case, extensive microbleeds and FLAIR hyperintense areas (edematous lesions) were observed in the cerebellum, right thalamus, and bilateral cortical and subcortical areas upon initial examination. Despite being asymptomatic, these findings indicated a high risk for future deterioration. Over time, the patient began experiencing a decrease in appetite and an increased tendency to fall two months later. Three months after the injury, she experienced a transient loss of consciousness, which led to a significant decline in functional status. This progression is thought to be related to the gradual damage to the subcortical neural networks caused by the inflammatory process and the brain dysfunction associated with vascular edema and microbleeds [7,8]. Additionally, follow-up MRI at six months showed resolution of the initial edematous lesions but revealed significant atrophy in the bilateral medial temporal lobes, suggesting post-inflammatory neurodegeneration, a finding that supports the potential for irreversible neuronal loss following inflammation in CAA-ri [9].

Once the diagnosis of CAA-ri is confirmed by biopsy, immunosuppressive therapy, including steroid pulse therapy, is generally recommended. In this case, brain biopsy was not performed, which reflects a common clinical challenge in elderly patients where the risks of an invasive procedure often outweigh potential diagnostic benefits. However, based on characteristic MRI findings and clinical presentation, the diagnosis of probable CAA-ri was made according to internationally accepted diagnostic criteria [3]. In a report by Auriel et al., untreated CAA-ri patients had worse functional outcomes compared to those who received treatment, emphasizing the importance of early intervention [3]. Early treatment may reduce the risk of relapse and improve prognosis [10]. When managing asymptomatic cases, it is important to carefully balance the benefits of treatment against the potential side effects of steroids (e.g., infections, worsening diabetes, osteoporosis). If the symptoms are mild and inflammation is localized, observation may be acceptable. However, in cases with widespread white matter edema, multiple CMBs, or cSS, even asymptomatic patients should be considered for active treatment [7,8]. In this case, although the patient was initially asymptomatic, the extensive imaging abnormalities suggested that a more aggressive immunosuppressive treatment could have been considered.

This case highlights that even patients with probable asymptomatic CAA-ri exhibiting extensive MRI abnormalities may carry a substantial risk of neurological deterioration and functional decline. In particular, patients with high-risk features, such as microbleed burden and widespread white matter edema, may require active management, regardless of symptom presence. Establishing management guidelines for asymptomatic cases will require a further accumulation of cases and prospective studies.

## Conclusions

This case report describes a rare instance of probable asymptomatic CAA-ri that was incidentally detected following head trauma and subsequently progressed to symptomatic disease, ultimately resulting in a decline in functional status. Although the patient was asymptomatic at initial presentation, brain MRI revealed extensive white matter edema and multiple microbleeds, highlighting a clear mismatch between clinical silence and the severity of imaging abnormalities.

Given the patient's advanced age and the substantial radiological burden, this case emphasizes the need for early risk stratification and a low threshold for considering immunosuppressive therapy, even in asymptomatic cases. Timely recognition and intervention may help prevent irreversible neurological decline. This report underscores the importance of individualized management and highlights the need for further studies to inform treatment strategies for asymptomatic CAA-ri.
